# Assessing the Social Value of Ecosystem Services for Resilient Riparian Greenway Planning and Management in an Urban Community

**DOI:** 10.3390/ijerph17093261

**Published:** 2020-05-07

**Authors:** Junga Lee, Byoung-Suk Kweon, Christopher D. Ellis, Sang-Woo Lee

**Affiliations:** 1 Department of Forestry and Landscape Architecture, Sanghuh College of Life Sciences, Konkuk University, 225 Life Sciences Building, Seoul 05029, Korea; archjung@konkuk.ac.kr; 2 Department of Plant Science and Landscape Architecture, University of Maryland, 2140 Plant Sciences Building, College Park, MD 20742, USA; kweonb@umd.edu; 3 Department of Plant Science and Landscape Architecture, University of Maryland, 2144 Plant Sciences Building, College Park, MD 20742, USA; cdellis@umd.edu; 4 Department of Forestry and Landscape Architecture, Sanghuh College of Life Sciences, Konkuk University, 303-1 Life Sciences Building, Seoul 05029, Korea

**Keywords:** cluster analysis, importance-performance analysis, perception, familiarity

## Abstract

Ecosystem services depend on the interrelation between people and the environment, and people are increasingly recognizing the social value of ecosystem services. Based on humans needs related to the values of ecosystem services, riparian greenways, properly planned and managed for resiliency, could provide great opportunities for social ecological change and transformation toward sustainability. We focus on the ecosystem service values of such greenways based on resilience in urban communities. The purpose of this study is to assess the social value of ecosystem services for resilient riparian greenway planning and management based on a survey of residents living near the Yangjaecheon riparian greenway in Gwacheon, South Korea. First, cluster analysis was performed with data from 485 completed surveys to identify different groups of respondents. Importance-performance analysis (IPA) was then applied to develop planning and management guidance for the riparian greenway based on group characteristics. Two distinct groups were identified: the Strong Social Value of Ecosystem Services group and the Neutral Social Value of Ecosystem Services group. Different distributions were found between the two groups based on gender and residency period, and significant differences were also found for age and familiarity with the riparian greenway. The results show what each group perceived to be important and how well the riparian greenway met their expectations regarding ecosystem services. These results indicate the perceived value of ecosystem services on the basis of the group characteristics, helping establish the direction for resilient riparian greenway planning and management approaches.

## 1. Introduction

Riparian greenways are vegetated linear corridors formed by the connection of two or more vegetation buffer strips that are established along a river [1]. Such greenways are based on natural resources [2], and they serve as the backbone of local and regional greenway networks [3], which are partly the result of a deep-rooted affinity that people have for watercourses in their communities. As a system of greenway networks, riparian greenways efficiently provide both tangible and intangible ecosystem services to enhance a community’s quality of life [4,5]. Fabos [6,7] suggested the following three objectives for greenways: (1) nature protection, because maintaining existing levels of biodiversity is the best investment people can make at the least cost to future generations; (2) maximizing recreation and tourism opportunities, because greenways can provide healthy environments where one can restore his or her sense of well-being and explore and satisfy a range of active and passive recreational needs and desires; and (3) protecting and restoring historical and cultural heritage, because greenways provide a viable path for these objectives. These three functions support a variety of ecosystem services [2,8,9,10,11,12,13,14]. All riparian greenways have certain basic characteristics in common, but the diversity of greenways causes them to function in different ways to provide different ecosystem services. According to the Millennium Ecosystem Assessment [15,16,17,18], the ecosystem services that riparian greenways provide are divided into provisioning services, regulating services, cultural services, and supporting services. For example, provisioning services, such as food (notably fish), fiber, and fresh water, are the essential products that are obtained from rivers and greenways to support human well-being. Regulating services, including air quality, climate, nutrient cycling, and water cycling regulation, are based on enhancing the quality of the natural environment. Cultural services are represented by the aesthetic, cultural, and spiritual value derived from environmentally related activities (e.g., recreation and tourism). In addition, supporting services, including biomass and atmospheric oxygen production and soil formation and retention, are necessary for the realization of all other ecosystem services [15,16,17,18]. These ecosystem services that riparian greenways provide are critical for sustaining vital ecosystem functions that deliver many benefits to people.

To manage riparian greenways sustainably in urban communities, the beneficiaries or the anticipated value of ecosystem services need to be understood. Urban communities are complicated social-ecological systems with multiple strong or weak interactions between components at various spatial and temporal levels and scales [19,20,21]. The development of resilient, equitable, and sustainable urban communities depends on the social-ecological systems’ ability to maintain both social and ecological functions at the same time [22]. Ecosystem services play an important role in interconnecting ecological components and social components in urban communities by providing benefits to humans and ecosystems [23]. By planning or managing various ecological and social components, especially in riparian greenways in urban communities, resilient systems can be maintained and managed to sustainably provide ecosystem services. The valuation of such ecosystem services can be a meaningful approach to identify the importance or performance of ecosystem services to beneficiaries in communities [24].

The valuation of ecosystem services can be a particularly useful indicator of human welfare and sustainability at the macro level [25]. Many studies have proposed frameworks that involve valuing and understanding people’s relations with place from a human welfare point of view (e.g., [25,26,27,28]). The social value of an ecosystem service is a result of human perceptions regarding the performance of a natural ecosystem [27,29,30,31,32] based on what is or is not considered to be important [33]. In other words, it can be quantified based on human perceptions regarding the importance of the ecosystem [30,34], and there are two types of social values: consumptive social value and nonconsumptive social value [35,36]. Consumptive social value includes provisioning, supporting, and regulating services such as irrigation and the public water supply. Nonconsumptive social value includes cultural services such as recreation and tourist activities, landscape and aesthetic aspects, and educational and scientific benefits. The social value of ecosystem services plays a role in identifying the importance of environmental functions and emphasizes physical and mental health, education, cultural diversity, heritage, and spiritual values [37]. The valuation of these services can be used to examine the relation between the social value of ecosystem services and the natural conditions in the underlying landscape. When developing landscape planning and management policies, decision makers can use knowledge on the social value of ecosystem services to understand stakeholders’ perceptions concerning the natural areas in their region [33,38]. In addition, using cluster analysis, stakeholders like residents can be segmented further into subgroups based on characteristics related to perceived social value of ecosystem services. This approach is important as it delivers the meaningful insight on landscape planning and management according to the residents’ unique needs [39,40]. Identifying and developing an understanding of meaningful resident subgroups will be an important first step for landscape planning and management policies in understanding this diverse residents’ perception and design messages from them. In addition, such an understanding of social value can increase the benefits of environmental management based on biophysical and economic value and enhance engagement according to stakeholders’ distinction in the development of planning strategies to address the spatial pattern of resiliency [41].

In the process of spatial planning for resilient systems, the social value of ecosystem services is an essential consideration for environmental sustainability and economic growth in social-ecological systems in human society [8,42]. Because the benefits of ecosystem services are associated with human well-being, it is generally believed that the social value of ecosystem services can be a useful indicator of the welfare of a community [25]. The social value of ecosystems can be the basis upon which to maintain urban communities that are resilient to systemic change while promoting human health and well-being through the design, planning, and management of urban communities as complex social-ecological systems. Some studies on landscape planning alternatives are based on residents’ perceptions concerning the social value of ecosystem services from the social-ecological system in urban communities [24,43,44,45]. Riparian greenways, in particular, are a type of multifunctional complex social-ecological system that can be used to incorporate ecosystem benefits into resilient landscape planning alternatives because of the provisioning of ecosystem services to enhance a community’s quality of life. Achieving resilient landscape planning and human welfare in riparian greenways as social-ecological systems in urban communities will require a better understanding of residents’ perceptions of riparian greenways in terms of ecosystem services.

The purpose of this study is to assess the social value of ecosystem services for resilient riparian greenway planning and management in urban communities. This study thus examines residents’ perception of the importance and performance of ecosystem services to assess the social value of ecosystem services provided by riparian greenways. The implications of the findings will be discussed to provide spatial planning and management guidance for the development of resilient riparian greenways according to the perceived social value of ecosystem services. This study can help planners and managers of riparian greenways understand the attitudes of users and thus identify directions for potential planning strategies based on the target users.

## 2. Materials and Methods

### 2.1. Study Site

**Figure 1:**
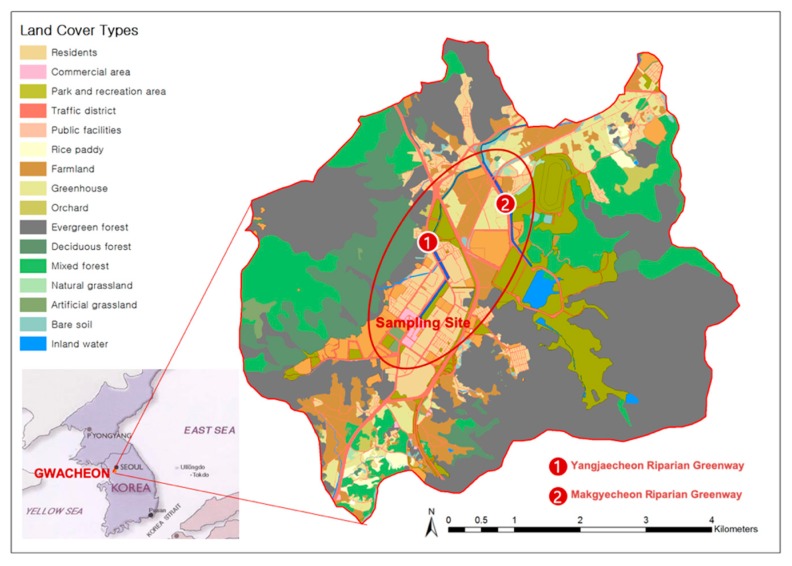
Study site.

For the purpose of this study, Gwacheon, a small- to medium-sized urban area near Seoul that is located in the midwestern region of Gyeonggi-do, Korea, with an approximate population of 71,000, was selected (Figure 1). Gwacheon is geographically situated at 37°23’53″ to 37°27’52″ N and 126°57’52″ to 127°02’52″ E. This study focused on the riparian greenways in Gwacheon: the Yangjaecheon and Makgyecheon greenways. The Yangjaecheon River, the main river in Gwacheon, flows from the southwest to the northeast in Gwacheon, extends for approximately 5.50 km^2^, and has a basin area of 37.21 km^2^. The Makgyecheon River flows from the southeast of Gwacheon to the northwest until it joins the Yangjaecheon River. The Makgyecheon basin area is 10.48 km^2^. Riparian trails were developed along both sides of the river, and hiking and biking are the most popular recreational activities in the area. The Yangjaecheon and Makgyecheon greenways have excellent usability because these rivers are easily accessible from downtown and residential areas.

### 2.2. Research Process

Here, we assess the social value of the ecosystem services provided by riparian greenways to generate discussion about how planning and management may be improved to allow riparian greenways to serve as a more resilient social-ecological system. This study thus examines residents’ perception of the importance and performance of the social value of ecosystem services provided by riparian greenways. Specifically, this study involved four steps:

(1) Classification of users into different groups based on their perceptions of the importance of ecosystem services;
(2) Examination of demographic information on and familiarity with riparian greenways to compare the group characteristics;
(3) Assessment of the differences between the perceived importance and the performance of the ecosystem services by using an importance-performance analysis (IPA); and
(4) Discussion of planning and management guidance for the development of resilient riparian greenways by focusing on each group.

This study can help planners and managers develop resilient riparian greenways through the understanding of the attitudes of users and thus identify directions for potential planning based on the target users.

### 2.3. Sampling Method

The study sample was the residents who live near the study site: the Yangjaecheon and Makgyecheon riparian greenways. The survey was delivered to each resident’s mailbox in a residential area in Gwacheon adjacent to the riparian greenways. The researchers contacted the representative of the residential area and were granted permission to deliver the survey sheets. The mail-back survey sheets were delivered to 2500 households in this residential area. This mailing included a cover letter that explained the purpose of the study and how to mail back the survey sheets in one week. A total of 512 individuals responded; thus, the response rate was 20.48%.

### 2.4. Research Instrument

The questionnaire used for this study included three main sections. The first section consisted of questions related to the social value of ecosystem services attributes. These attributes were identified from Reed and Brown [6] based on a review of the relevant literature. Reed and Brown [6] developed and proposed 12 typologies of the social value of ecosystem services that can be used as indicators to measure and predict stakeholder attitudes concerning ecosystem benefits (Table 1). These social value types have been applied in numerous community-based surveys [9,10,11,46,47]. Using these social value types, in this study, the residents were asked to indicate the perceived importance of the attributes of a riparian greenway and their perceptions of the actual performance of the riparian greenway in Gwacheon. A 5-point Likert scale was used to measure the importance and performance of the social value of the ecosystem services. For importance, the scale ranged from 1—not important at all to 5—very important, and for performance, the scale ranged from 1—very poor performance to 5—very good performance.

The second part of the questionnaire was designed to investigate the visitation patterns and familiarity with the study site. To measure familiarity with the riparian greenway of Yangjaecheon, the scale items were developed based on our literature review. According to several studies, familiarity is influenced by the level of exposure to information, the degree of intimacy, and the frequency of interaction [48,49,50]. Therefore, the familiarity dimension consists of the five following statements: “I feel a sense of pleasantness toward the riparian greenway of Yangjaecheon”; “I often see information about the riparian greenway of Yangjaecheon in my surroundings”; “I often talk with friends about the riparian greenway of Yangjaecheon”; “I know much about the riparian greenway of Yangjaecheon”; and “I am familiar with the riparian greenway of Yangjaecheon because my friends and I often visit it.” The respondents were also asked to rate these statements according to a Likert-type scale of from 1 to 5, where 1—strongly disagree and 5—strongly agree.

The third part of the questionnaire included questions related to sociodemographic data, including the residency period. The residents’ responses to the questions in the last two parts were used to identify the segments.

A preliminary survey was conducted as a pilot test to verify the reliability of all variables and the questionnaires. After the instrument development process, a series of on-site surveys was performed near the study site using a random sampling method. To investigate the reliability of the survey instrument, the collected data were analyzed using descriptive statistics and a reliability test. All data were analyzed using IBM SPSS Statistics 12.0 (SPSS Inc., Chicago, IL, USA). A total of 85 (90.4%) respondents provided useful data after the removal of 9 invalid questionnaires. The respondents’ average age was 41.93, and 71.8% of the respondents lived in Gwacheon. The collected data were analyzed using descriptive statistics. Reliability was also assessed for the importance of social values (Cronbach’s α = 0.782), performance of social value (Cronbach’s α = 0.874), and familiarity (Cronbach’s α = 0.861).

### 2.5. Data Analysis

The data were analyzed using descriptive statistics, cluster analysis, IPA, and t-tests with IBM SPSS Statistics 12.0 (SPSS Inc., Chicago, IL, USA). The demographic information was evaluated using descriptive statistics. Descriptive statistics were also used to assess the perceived importance and performance of riparian greenways in terms of the social value of ecosystem services. After the descriptive data were presented, a reliability test was performed for each variable. The reliability test indicated that all variables were highly reliable for further analysis (the Cronbach alpha coefficients for the 12 variables associated with ecosystem services = 0.859).

Subsequently, cluster analysis was used to classify the respondents into different groups based on the perceived importance of ecosystem services. A hierarchical clustering procedure was adopted to group the residents, with the Euclidean distance between the cases used as a similarity measure. The clustering procedure included the K-means clustering method to determine the best number of clusters based on the importance attributes of the social value of ecosystem services.

IPA was performed to compare the perceived importance and performance of the riparian greenways to understand the social value of ecosystem services based on the clusters. These clusters of residents were compared based on the different constructs related to the familiarity and visitation pattern data and that of the demographic categories. In addition, t-tests were used to identify the mean differences among the cluster groups. Chi-square tests were also performed with the cluster and classification data. The next step applied IPA grids to each cluster of residents. Finally, a t-test in support of the IPA was used to determine whether a significant difference existed between the perceived importance and performance relative to the list of variables related to the social value of ecosystem services.

**Figure 2:**
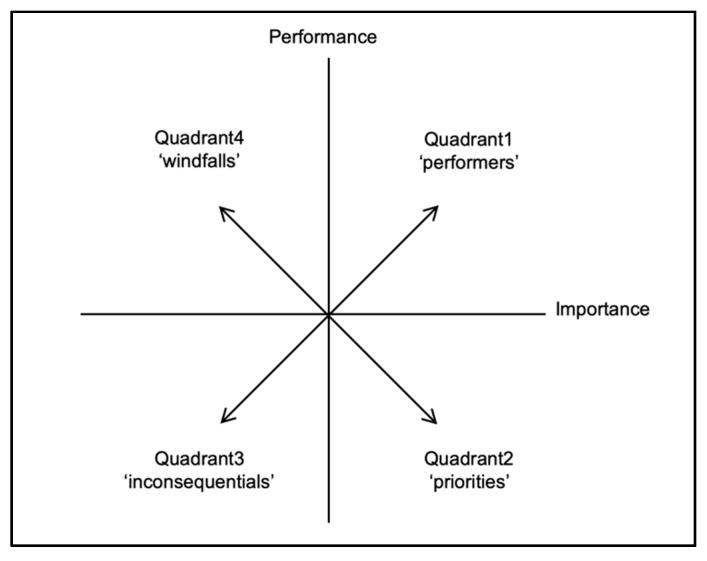
Schematic of the importance-performance analysis (IPA) grid.

IPA is a technique that can be used with certain attributes to analyze customer satisfaction based on the expectations of a product’s or service’s performance [51]. The IPA technique has been applied to analyze the strengths and weaknesses of products, services, brands, and retail establishments in various fields, as well as in the environmental issues. It is a broadly applicable tool that can be used to effectively assess the value of the services derived from an ecosystem. In IPA, the mean values of importance and performance provide plot points for an IPA grid as a measure of central tendency [52]. The IPA grid is constructed by using a horizontal (x) axis and a vertical (y) axis. The horizontal (x) axis represents the degree of importance scores, whereas the vertical (y) axis represents the degree of performance scores. Each of the four quadrants in the IPA grid represents considerations for planning and management to enhance the social value of ecosystem services. The issues of importance to residents and the areas where greenways perform well will fall within the upper right quadrant of the grid. Thus, landscape planning and management policymakers can identify these items as “Performers.” These items act as indicators of how a riparian greenway can best meet the concerns regarding the social value of ecosystem services. The items that are considered important to the social value of ecosystem services but for which the riparian greenway does not perform well fall within the lower right part of the grid. These items require more attention in future planning and management efforts. The items in this area may be labeled “Priorities.” The issues of low importance but for which the riparian greenway is considered to perform well fall within the upper left quadrant and may be considered “Windfalls.” Finally, certain items may be perceived as unimportant and receive low performance scores. These items are apparently of little concern, and the fact that a riparian greenway does not contribute to them may not matter much. These items are located within the lower left part of the grid and may be labeled “Inconsequentials” (Figure 2).

## 3. Results

### 3.1. Demographic Characteristics of the Respondents

The analysis used data from the 488 completed surveys of the 512 collected surveys. Table 2 shows the demographic characteristics of the respondents. The sample did not perfectly reflect the population of interest but approximately reflected it. The sample was 61.8% female (n = 301), and the population was approximately 51.3% female. The demographic breakdown of the respondents indicates that the largest percentage was people in their 40s (20–29, 16.5%; 30–39, 15.3%; 40–49, 31.2%; 50–59, 16.5%). Most respondents were highly educated, and the majority held a college/university degree (54.7%). When their occupational status was compared, the largest group comprised housewives (30.4%), followed by students (19.5%). On average, the respondents had lived in the area for 10.79 years.

### 3.2. Classification of Respondents Based on the Importance of the Social Value of Ecosystem Services

After evaluating the overall importance of the social value of ecosystem services, we determined how the residents could be meaningfully divided into different groups. We also assessed how they differed in their perceptions of the degree of importance of the different variables that measure the social value of the ecosystem services provided by riparian greenways. The cluster analysis showed that two groups (clusters) were appropriate for the data based on the residents’ perceptions of the importance of the social value of the ecosystem services variables. The t-test results also indicated that the 12 attributes contributed to differentiating the two clusters (*p* < 0.001). These clusters were labeled as the Strong Social Value of Ecosystem Services group (S-SVES group) (n = 267, 54.7% of the sample) and the Neutral Social Value of Ecosystem Services group (N-SVES group) (n = 221, 45.3% of the sample), as shown in Table 3. The S-SVES group perceives a greater importance of ecosystem services than the N-SVES group.

### 3.3. Importance-Performance Analysis of the Riparian Greenway

**Figure 3:**
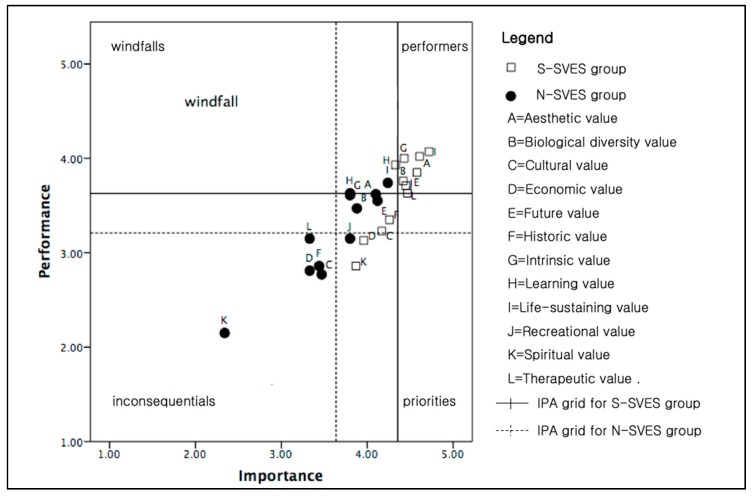
IPA for each cluster.

The social value of the ecosystem services variables was plotted based on both the importance that people placed on the variables and how well they felt that the riparian greenway performed. The mean values of the social value attributes for both the importance and performance of the riparian greenway were plotted in an IPA grid. The quadrants were created by averaging the mean values of all importance and performance responses. Figure 3 shows the IPA results for the entire population comprising the S-SVES and N-SVES groups.

The mean values of the response scale for the S-SVES group were higher than those for the N-SVES group. The 12 types of ecosystem service values are plotted on the IPA grid for each group. Both groups have similar perceptions of the “Aesthetic”, “Biological”, “Future”, “Intrinsic”, and “Life-sustaining” values, which are all represented in the “Performers” quadrant as high performers. “Cultural”, “Economic”, “Historic”, and “Spiritual” values are represented in the “Inconsequentials” quadrant for both groups. However, the “Learning”, “Recreational”, and “Therapeutic” values show different perceptions for the S-SVES and the N-SVES groups. In the IPA of the S-SVES group, the “Learning” value was represented in the “Windfall” quadrant. Conversely, in the IPA of the N-SVES group, the “Learning” value was represented in the “Performers” quadrant. Concerning “Recreational” value, the S-SVES group believes that the riparian greenway is a good performer. However, the N-SVES group believes that the “Recreational” value is important but that the riparian greenway does not meet its performance expectations. The S-SVES group also believes that the riparian greenway is a good performer in terms of the “Therapeutic” value. In contrast, the N-SVES group has little concern for the “Therapeutic” value; the riparian greenway does not contribute to this category, and the greenway may not matter much to this group.

### 3.4. Differences between the S-SVES Group and the N-SVES Group

To provide additional information differentiating the two groups, a chi-square analysis was performed on the clusters based on the five demographic characteristics. As shown in Table 4, there was a significant difference in the distribution by gender in the two clusters. Although female participation was high in both groups, there was a considerably higher female percentage (67.0%) in the S-SVES group and a higher male percentage (44.8%) in the N-SVES group. There was also a significant difference in the distribution by residency period in the two clusters. There were more residents (24.3%) with over 20 years of residency in the S-SVES group than in the N-SVES group. In addition, an independent-samples t-test showed a significant difference in age (*p* < 0.05) between the S-SVES group (mean = 42.17 years, std. deviation = 14.154) and the N-SVES group (mean = 38.60, std. deviation = 15.308). An independent t-test was used to investigate the differences in familiarity with the riparian greenways between the S-SVES group and the N-SVES group (Table 4). The results revealed that the S-SVES group (mean = 3.280) had a significantly higher familiarity than the N-SVES group (mean = 2.908).

## 4. Discussion

This study demonstrates that IPA can be used as an effective tool to develop resilient riparian greenway management and planning policies for different population groups; this study also shows how IPA can help to prioritize riparian greenway issues based on nearby residents’ reported values. A close inspection of the ratings for each individual IPA variable provides an effective way for planners and managers of greenways to assess residents’ perception of greenway performance. This inspection indicates residents’ priorities and whether the greenway is functioning sufficiently well to meet their expectations on each performance measure.

The perceived importance and performance of the social value of ecosystem services can also differ across groups, and these perceptions can help decision makers develop a spatial planning process [33,38,41]. The value of ecosystem services is assigned based on the perceived qualities of the environment that provides benefits to people [53,54,55]. Thus, the relative perceived importance of the social value of ecosystem services can provide value-related concepts to help strengthen landscape planning and management policy based on human–environment interactions. The spatial pattern of the plots shows that the values of consumptive ecosystem services were in the good, better, and best categories of performers, in contrast to the values of nonconsumptive ecosystem services.

Specifically, one concern regarding the planning and management of the riparian greenways is that the two groups share the same beliefs in terms of consumptive and nonconsumptive ecosystem services. Both groups thought the riparian greenway was a good performer in terms of consumptive ecosystem services, such as the “Aesthetic”, “Biological”, “Future”, “Intrinsic”, and “Life-sustaining” values. However, both groups thought that nonconsumptive ecosystem services were low priorities, including the “Cultural”, “Economic”, “Historic”, and “Spiritual” values. Consumptive ecosystem services incorporate biophysical or ecological services from the ecosystem functions of riparian greenways. From these consumptive ecosystem services, nonconsumptive ecosystem services can be created based on the demand for ecosystem services from a sociocultural viewpoint by examining the importance that people place on particular services. Until recently, many aspects of the landscape planning and management process have focused on consumptive ecosystem services that are perceived by society to be most important; thus, this process does not fully encompass the tradeoffs with nonconsumptive ecosystem services. Such ecosystem service tradeoffs arise when certain ecosystem services are reduced because of the increased use of another ecosystem service [56]. Similarly, riparian greenways, such as the study site, do not fully capture the value of nonconsumptive ecosystem services, in contrast with the value of consumptive ecosystem services.

The other concern involving the planning and management of riparian greenways based on user targets is that the two groups have different perceptions regarding the “Learning”, “Recreational”, and “Therapeutic” values. Because the S-SVES group has a higher familiarity than the N-SVES group, a high frequency of exposure to and the usability of the riparian greenways can have a positive effect on enhancing user satisfaction. Conversely, because the N-SVES group has lower familiarity than the S-SVES group, even if it has high satisfaction with the riparian greenways, such as a high “Learning” value, the degree of perceived importance cannot be fully explained. In terms of “Recreational” value, a lower frequency of visits may have an influence on the lower perceived performance for the S-SVES group than for the N-SVES group. Accordingly, landscape planners should engage in sustainable efforts and provide a variety of attractions and user opportunities that relate to recreation and education to encourage demand for riparian greenways from users and stakeholders. Additionally, the “Therapeutic” value would not be well known to the N-SVES group. According to the characteristics of the two groups, using IPA can also help to provide a decision-making process for landscape planning and management strategies that focuses on specific user targets. For example, considering the “Learning” value’s position in the IPA grid, the distribution of the demographic information in the N-SVES group signals that educational programs communicating the value of ecosystem services provided by riparian greenways to relatively young users should be offered throughout the landscape planning and management process.

The demographic variables of the respondents suggest a need to more closely examine place familiarity with the riparian greenways by clusters. In general, place familiarity refers to the degree of indirect and direct experience with and information that is known concerning the place that is visited [57]. According to the literature review, familiarity is influenced by the level of information exposure, the degree of intimacy, and the frequency of interaction [48,49,50]. We found that the S-SVES group perceived more familiarity than the N-SVES group. This result may be affected by the distribution of demographic information in each group, such as age, job, and residency period.

## 5. Conclusions

In this study, a cluster analysis identified two groups of residents who live near the riparian greenway as having strong ecosystem services values (S-SVES group) and neutral ecosystem services values (N-SVES group) based on the reported social values of ecosystem services. This study attempted to identify both the importance and performance of the social value of the ecosystem services in the riparian greenway using an IPA. According to the IPA, the “Aesthetic”, “Biological”, “Future”, “Intrinsic”, and “Life-sustaining” values were all found to be important and performing well. For the S-SVES group, however, the “Learning” value was not as important but was performing as well as the other value categories. This result suggests that less effort can be exerted toward educational activities and promotional materials for the S-SVES group. Other areas, such as the “Cultural”, “Economic”, “Historic”, and “Spiritual” values, were reported to be less important and performing at a lower level. If these low IPA perceptions are not consistent with the goals of the greenway’s management, then additional resources may be needed to boost the perception of the importance and performance of these values among the area’s residents. The “Recreational” and “Therapeutic” values both tended toward the intersection of the IPA quadrants for both the S-SVES and N-SVES groups. These values are neither the highest- nor lowest-ranked variables in terms of importance or performance. Recreation is clearly an important goal for greenways. This finding suggests that the residents are generally satisfied with the Yangjaecheon greenway as a place for recreation. The same is true for the therapeutic qualities of the greenway. Although many approaches are available to measure the environmental and biological performance of greenways, fewer methods are available to measure their social performance. IPA offers an effective and flexible tool to evaluate local residents’ perceptions about the functioning of greenways along a number of important social dimensions.

This study also examined the differences between the two groups based on their demographic information and their familiarity with the study site. The S-SVES group placed higher importance on the social value of the ecosystem services variables. This group was composed of a higher percentage of older female residents reporting significantly higher familiarity with the Yangjaecheon greenway than the N-SVES group. The N-SVES group placed lower importance on the social value of the ecosystem services variables. Greenway managers can use the information obtained through cluster analysis to target certain residents for educational and promotional information, such as the N-SVES group, which reported lower importance and performance scores in general than the S-SVES group. Greenway managers can also use this information to recruit willing volunteers or provide special services and activities to residents who highly value riparian greenways, such as the S-SVES group.

The results of this study show that recognizing the value of ecosystem services according to group characteristics can help set the direction for riverside greenway planning and management, which is the basis for maintaining a resilient riparian greenway.

## Figures and Tables

**Table 1:** Types of social values of ecosystem services.

Type	Description
Aesthetic value	I value the place because I enjoy the natural scenery, sights, sounds, smells, etc.
Economic value	I value the place because it provides timber, fish, minerals, or tourism opportunities such as outfitting and guiding.
Recreational value	I value the place because it provides a space for my favorite outdoor recreational activities.
Life-sustaining value	I value the place because it helps produce, preserve, clean, and renew air, soil, and water.
Learning value	I value the place because we can learn about the environment through scientific observation or experimentation.
Biological diversity value	I value the place because it provides a variety of fish, wildlife, plant life, etc.
Spiritual value	I value the place because it is a sacred, religious, or spiritually special site to me or because I feel reverence and respect for it.
Intrinsic value	I value the place in and of itself for its existence no matter what I or others think about it.
Historic value	I value the place because it contains places and things associated with natural and human history.
Future value	I value the place because it allows future generations to know and experience the place as it is now.
Therapeutic value	I value the place because it makes me feel better, physically and/or mentally.
Cultural value	I value the place because it is a place where I can continue and pass down the wisdom, knowledge, traditions, and way of life of my ancestors.

Source: Brown and Reed [35].

**Table 2:** Sociodemographic profile of the respondents.

Attribute		n	%
Gender	Male	186	38.2
	Female	301	61.8
Age	10–19	51	10.5
	20–29	80	16.5
	30–39	74	15.3
	40–49	151	31.2
	50–59	80	16.5
	60–69	31	6.4
	70–79	14	2.9
	80–89	3	0.6
Educational background	Below high school	114	23.7
	Community college degree	49	10.2
	College degree	263	54.7
	Graduate/professional	55	11.4
Job	Management	11	2.3
	Public officer	32	6.7
	Engineer	14	2.9
	Office work	46	9.6
	Own business	29	6.0
	Profession	41	8.5
	Housewife	146	30.4
	Sales position	13	2.7
	Retired/Unemployed	10	2.1
	Student	94	19.5
	Other	45	9.4
Residency period (mean)		10.79 years	

**Table 3:** Differences between the two clusters of respondents.

Attribute	S-SVES Group ^1^ (n = 267)		N-SVES Group ^2^ (n = 221)		t-Value	df	*p*-Value
	Mean	Std. Deviation	Mean	Std. Deviation			
Aesthetic value	4.610	0.546	4.100	0.546	9.489	486	0.000
Biological diversity value	4.420	0.718	3.880	0.642	8.197	467	0.000
Economic value	4.170	0.761	3.470	0.723	10.248	486	0.000
Cultural value	3.960	0.824	3.330	0.754	8.206	486	0.000
Future value	4.580	0.565	4.120	0.851	7.664	410	0.000
Historic value	4.260	0.735	3.440	0.725	11.308	439	0.000
Intrinsic value	4.430	0.671	3.800	0.844	9.546	486	0.000
Learning value	4.330	0.692	3.800	0.796	8.356	486	0.000
Life-sustaining value	4.720	0.492	4.240	0.711	8.609	387	0.000
Recreational value	4.470	0.609	3.800	0.689	10.499	411	0.000
Spiritual value	3.870	0.843	2.340	0.780	18.491	442	0.000
Therapeutic value	4.450	0.626	3.330	0.957	19.293	486	0.000

^1^ S-SVES group: Strong Social Value of Ecosystem Services group; ^2^ N-SVES group: Neutral Social Value of Ecosystem Services group.

**Table 4:** T-test of familiarity by cluster.

Attribute	S-SVES Group (n = 267)		N-SVES Group (n = 221)		T-Value	df	*p*-Value
	Mean	Std. Deviation	Mean	Std. Deviation			
Total familiarity	3.280	0.769	2.908	0.719	5.473	483	0.000
I feel a sense of friendliness toward the riparian greenway of Yangjaecheon.	3.940	0.795	3.520	0.190	5.777	470	0.000
I often see information about the riparian greenway of Yangjaecheon in my surroundings.	3.170	0.942	2.860	0.886	3.748	486	0.000
I often talk with friends about the riparian greenway of Yangjaecheon.	2.940	1.041	2.600	0.951	3.805	486	0.000
I know the riparian greenway of Yangjaecheon well.	3.090	0.939	2.740	0.836	4.214	483	0.000
I have familiarity with the riparian greenway of Yangjaecheon because my friends often visit it.	3.260	1.037	2.820	1.032	4.628	485	0.000
